# Supplementary material for: Management of plant nutrient dynamics under alkaline soils through graded application of pressmud and gypsum
Source: PLoS One. 2023 Aug 9;18(8):e0288784. doi: 10.1371/journal.pone.0288784 (PMC10411785; doi:10.1371/journal.pone.0288784)
Supplement: Attachment — Submitted filename: Manuscript.doc [file pone.0288784.s001.doc]

**Management of Plant Nutrient dynamics under Alkaline Soils through graded application of Pressmud and Gypsum**

*Subtitle: Management of Alkaline soils by addition of pressmud and Gypsum*

ML Dotaniya^1,*^, MD Meena^1^, RL Choudhary^1^, MK Meena^1^, Harvir Singh^1^, CK Dotaniya^2^, LK Meena^1^, R K Doutaniya^3^, KN Meena^1^, RS Jat^1^, PK Rai^1^

^1^ICAR-Directorate of Rapeseed- Mustard Research, Bharatpur-321 303, India

^2^Department of Soil Science & Agricultural Chemistry, SKRAU, Bikaner-334 006, India

^3^Department of Agronomy, SKN College of Agriculture, Jobner-303329, India

*corresponding author e mail; [mohan30682@gmail.com](mailto:mohan30682@gmail.com) (ML Dotaniya); [murliiari@gmail.com](mailto:murliiari@gmail.com) (MD Meena)

ORCID ID. orcid.org/0000-0001-9311-088X

Phone: +91-5644-260419; Fax: +91-5644-260565

**ABSTRACT**

An incubation experiment was conducted to monitor the effect of different organic matter inputs with the graded application of gypsum at different time intervals on soil pH, sodium (Na) content and available plant nutrients like nitrogen (N) and sulphur (S). The experiment was formulated with nine treatments, *i.e.* control (T_1_), recommended dose of fertilizer (RDF) (T_2_), RDF+Gyp_1_ (T_3_), RDF+FYM_5_+Gyp_2_ (T_4_), RDF+FYM_10_+Gyp_1_ (T_5_), RDF+PM_5_+Gyp_2_ (T_6_), RDF+PM_10_+Gyp_1_ (T_7_), RDF+FYM_2.5_+PM_2.5_+Gyp_2_ (T_8_), RDF+FYM_5_+PM_5_+Gyp_1_ (T_9_) with three replications. Periodical soil samples were taken at six and twelve months intervals. Results showed that the addition of organic matter reduced the pH and Na content in the soil. More reduction was observed at one year period as compared to six months. The addition of FYM and PM at 10 t/ha improved available N from 175 to 235 kg/ha; whereas, available S content from 15.4 to 23.2 kg/ha in soil. Pressmud (PM) application showed at par and better availability of plant nutrients and a reduction of soil pH (8.39 to 7.79) and Na content from 626 to 391 *m*Eq/L in the soil during the incubation period. During the study, the application of treatment T_9_ (FYM and PM in equal ratio with 1 t/ha gypsum) showed a better availability of available N (175 to 235 kg/ha) and S (15.44 to 23.24 kg/ha) and reduced the active ion concentration of Na. This study is very useful for the management of sodium toxicity, improving soil health and the mineralization rate of organic matter through the application of PM for sustainable crop production.

**Keywords** Alkaline soils, Gypsum; Plant nutrient dynamics; Pressmud, Sodium content

**Abbreviations**

RDF	Recommended dose of fertilizer

FYM	Farm yard manure

Gyp	Gypsum

PM	Pressmud

MSW	Municipal solid waste

N	Nitrogen

S	Sulphur

Na	Sodium

CRD	Completely randomized design

m ha	Million hectares

mt	Million tonnes

EC_e_	Extract electrical conductivity

**INTRODUCTION**

Plant nutrients are critical to crop productivity, and nutrient application must be balanced for long-term agricultural production and soil health. The physico-chemical features of the soil and management parameters influence nutrient availability in the soil [1]. Higher concentrations of salty and sodic ions like chloride (Cl), sodium (Na), calcium (Ca), and associated carbonate and bicarbonate salts limit the availability of important plant nutrients [2]. In India's dry and semi-arid regions, the problem is exacerbated significantly. Due to a lack of organic matter in the soil, the application of plant-available nutrients throughout the crop season does not produce the expected results. All soil microbial activity is based on organic matter, which improves the mineralization kinetics of given inputs. About 6.74 million hectares of land in India are affected by salt, with a preponderance in particular places, such as the agriculturally significant Indo–Gangetic basin [3]. Sodic soils dominate roughly 3.77 million hectares, resulting in an annual loss of about 11.2 million tonnes (mt) of farm productivity valued at US$ 2.3 billion [4]. In saline/alkaline soils, salt ions impede the mineralization process and diminish the microbial population and diversity [5]. Under saline -sodic soils, crop plants have a limited nutrient supply capacity, according to researchers. It also said that environmental services were poor due to harsh climatic and soil conditions. Food production may be managed scientifically and rationally without harming the environment or human health [6].

By 2050, India's population could exceed 1.6 billion people, requiring 333 mt of food grain production to feed the hungry. Fresh water availability per capita is declining, from 4000 m^3^ in 1947 to 1683 m^3^ in 2021 [7]. Farmers are obliged to utilize low-quality water for irrigation, resulting in soil contamination with heavy metals, organic pollutants, and salt [8]. This is one of the most significant obstacles to crop output and long-term sustainability [7]. Many examples can be found throughout history that indicate how the usae of low-quality water exacerbated infertility and reduced crop productivity. The partial pressure of water is increased by salt ions, which limits water uptake from the soil to the plant. This process was also responsible for soil microorganisms' poor growth and survival. The addition of organic and inorganic components to saline- sodic soils is the need of the hour for long-term crop development [9].

The addition of organic matter to soil increased soil organic carbon, which is necessary for plant nutrient kinetics and soil biota growth [10]. Soil microbial organisms feed on soil carbon and are responsible for the rapid breakdown of organic materials due to an increased population. Different types of organic acids were released during the decomposition process. These features are mediated by soil chemical properties and improve soil aggregation [11], plant nutrients [12], soil conditions [13], and overall soil health. According to Dotaniya et al. [14], adding pressmud to sewage-irrigated soils reduced heavy metal labile fractions while increasing plant nutrient availability. Meena et al. [6] found that adding municipal solid waste (MSW) compost to soil increased the available S level and reduced salt ion toxicity in a similar way. Sugarcane manufacturers produce pressmud as a byproduct. Its potential usage in crop production systems is under- utilized in the agricultural segment [11]. It contains a lot of organic C as well as other plant nutrients. A trace amount of sugar increased the microbial population in the soil, speeding up the organic matter mineralization kinetics [15]. Farm yard manure (FYM) is an oldage agricultural feed that is used to boost soil organic carbon levels [16]. The majority of Indian soils have low to moderate levels of soil organic carbon, which is one of the key drivers of plant nutrient utilization efficiency [17]. Higher levels of organic matter must be added to saline-sodic soils in order to boost yield. In saline soil, FYM increased the microbial population and crop output [6]. The supply of FYM is dwindling with the passage of time, necessitating the use of an alternative source of organic matter. PM could be a reasonable alternative in this situation. The use of PM with the graded application of gypsum for the management of sodic soils was practiced.

In this backdrop, a hypothesis was formulated to monitor the effect on organic matter addition (source and levels) on sodic ions and availability of major plant nutrient in sodic soils.

**MATERIALS AND METHODS**

Experimental details

Geo-referenced bulk soil samples were collected from nearby Kumher village of Bharatpur district, India. It is located 27.32°N and 77.37°E. In Bharatpur, the summers are scorching hot while the winters are lovely. The temperature varies between 38°C and 45°C during the summer months of March to June. The temperature drops to roughly 27°C as the monsoon season (July–September) begins, with a humidity level of 70–75%. It is categorized under sodic soil by the analysis of soil parameters. Soil pH was measured by saturation soil paste and extracted electrical conductivity as mentioned in Singh et al. [18]. The analyzed data showed pH (8.42), EC_e_ (13.92dS/m), CO_3_^2-^ + HCO_3_^-^ was (11.09 *m*Eq/L), SO_4_^2-^ (114.3 *m*Eq/L), Ca^2+^ + Mg^2+^ (41.23 *m*Eq/L) and Na^+^ ions (632 *m*Eq/L) in soil. Soil plant nutrients like available N P K and S was measured as per the standard protocol mentioned in Singh et al. [18]. Most of the major plant nutrients were present in low to medium range of soil fertility. FYM was collected from the research farm of ICAR-DRMR, Bharatpur and PM was collected from the Daurala Sugarmil, Meerut. The PM and FYM utilized in the research were also examined by standard analytical procedures and found that PM properties like pH (8.32) EC (3.21), sodium content (0.014 %), potassium concentration (0.037 %), OC (9.55 mg/kg), total N (0.019%), total P (0.012 mg/kg) and S (0.059 %); whereas, FYM properties pH (7.24) EC (1.63), sodium content (ND), potassium concentration (0.042%), OC (14.09 mg/kg), total N (0.54%), total P (0.023 mg/kg) and S (0.012%). With the analysis data of soil samples and organic amendments; different treatment combinations were formulated by addition of FYM, PM and gypsum, *i.e.* control (T_1_), RDF (T_2_), RDF + Gyp_1_ (T_3_), RDF + FYM_5_ + Gyp_2_ (T_4_), RDF + FYM_10_ + Gyp_1_ (T_5_), RDF + PM _5_ + Gyp_2_ (T_6_), RDF + PM_10_ + Gyp_1_ (T_7_), RDF + FYM_2.5_ + PM_2.5_ + Gyp_2_ (T_8_), RDF + FYM_5_ + PM_5_ + Gyp_1_ (T_9_). Bulk soil was filled into a plastic pot (500 g in each) and added different treatments as mentioned above. Soil moisture (at field capacity) and temperature (28 ± 1°C) was maintained at laboratory conditions. Addition of distilled water based on the water loss at a time interval. Periodical destructive soil samples were taken at six and one year time interval. After processing soil samples, different properties of soil like pH, sodium content, available plant nutrients like phosphorus [19], available S [20]; and available soil N content was measured by Subbiah and Asija [21] as mentioned in Singh et al. [18].

*Statistical Analysis*

The different parameters were analysed and computed in various parameters data. These treatments were statistically analyzed in completely randomized design (CRD) with three replications as per method described in Gomez and Gomez [22].The study of variance method (ANOVA) was used to assess the significance of treatment effects. At the 0.05 level of significance, the treatment means were evaluated using the least significant difference (LSD).

**RESULTS AND DISCUSSION**

*Effect of soil amendment on soil pH*

Application of FYM, pressmud and gypsum showed a significant effect on soil pH (Table 1). At six months interval of control, the plots treated with recommended dose of fertilizer (RDD) only showed lower pH by the application of FYM at 5 t/ha with 2 t/ha gypsum application. However further increasing FYM levels 5 to 10 with 1 ton gypsum application reduced pH value 7.92 to 7.86, which was found at par with . A similar pattern, the addition of pressmud at 5 t/ha along with RDF and gypsum at 2 ton/ ha reduced the soil pH with further modification in PM application rate (PM increased 5 to 10 t/ha) and gypsum (gypsum 1 ton/ha). The addition of organic matter through the FYM at 5 ton/ha and PM at 5 t/ha with one ton /ha gypsum reduced the maximum soil pH. Among the comparison of all the treatments, the application of the higher amount of organic matter act as a powerful soil pH reducer. FYM and PM both are equally performing with respect to soil pH value. One year after the incubation period soil samples were analyzed and showed that the addition of a higher amount of FYM from 5 t/ha to 10 t/ha reduced the soil pH from 7.92 to 7.85; whereas, PM application rate from 5 to 10 t/ha with graded application of gypsum reduced more pH units (7.94 to 7.80). Maximum pH was reduced under treatment T_9_ (RDF+FYM_5_+PM_5_+Gyp_1_) and *at par* with the treatment T_8_ (RDF+FYM_2.5_+PM_2.5_+Gyp_2_) during the experiment. The mean value of the experiment showed that higher pH was under RDF and increasing the gypsum and organic matter graded application reduced the soil pH. Both gypsum and organic matter decline the pH but organic matter was influenced more that gypsum application. The addition of organic matter in soil enhances the microbial population and produce different type of organic acids which mobile the plant nutrient by reducing the soil pH. The addition of FYM at 10 t/ha reduced the soil pH and enhanced the crop yield. Different types of soil micro-organisms is reduced salt toxicity and improve plant nutrient dynamics [23]. This sort of increased EC is possible, because the decomposition processes of organic matter favor the accumulation of carbon di oxide (CO_2_) and release of large amount of salts in solution which results in higher EC [24]. Kalaivanan and Hattab [12] reported that pressmud application at 5 t/ha did not affect the soil pH and electrical conductivity (EC) did not show any marked variation with the application of enriched pressmud compost. Prabhavathi and Parama [25] reported that the addition of pressmud at 10 t/ha along with recommended dose of fertilizer (RDF) reduced the soil pH under finger millet crop. Sheoran et al. [26] reported that pressmud application (10 Mg ha^−1^) led to appreciable reductions in soil pH (1.6–3.6%) and exchangeable sodium percentage (ESP; 10.4–20.1%) with concomitant improvements in plant physiological and yield-related traits across different alkali soil.

**Table 1** Effect of FYM, pressmud and gypsum application on soil pH

| Treatment | Duration | | Mean |
| --- | --- | --- | --- |
|  | 6 Months | 12 Months |  |
| Control | 8.39^a^ | 8.43^a^ | 8.41 |
| RDF | 8.39^a^ | 8.43^a^ | 8.41 |
| RDF+Gyp_1_ | 8.03^b^ | 7.94^b^ | 7.99 |
| RDF+FYM_5_+Gyp_2_ | 7.92^bc^ | 7.92^b^ | 7.92 |
| RDF+FYM_10_+Gyp_1_ | 7.86^cd^ | 7.85^bc^ | 7.86 |
| RDF+PM _5_+Gyp_2_ | 8.04^b^ | 7.94^b^ | 7.99 |
| RDF+PM_10_+Gyp_1_ | 7.88^cd^ | 7.80^bcd^ | 7.84 |
| RDF+FYM_2.5_+PM_2.5_+Gyp_2_ | 7.82^cd^ | 7.77^cd^ | 7.80 |
| RDF+FYM_5_+PM_5_+Gyp_1_ | 7.79^d^ | 7.70^d^ | 7.75 |

Note: Treatment means in a column with the letters in common are not significant by Duncan’s New Multiple Range Test at 5% level of significance.

*Effect of soil amendment on soil sodium content*

The addition of FYM and pressmud in a combination of graded dose of gypsum was very much affected by the duration and soil amendments. Increasing the application rate of FYM upto 5 t/ha along with 2 t/ha gypsum reduced the sodium content (598 *m*Eq/L ) in soil but it is not significant (p<0.05) at six month time interval (Table 2). Increasing the FYM upto 10 t/ha with 1 t/ha reduced significant (p=0.05) amount of sodium (514 *m*Eq/L) over control, T_2_ (RDF), T_3_ (application 1 t/ha) and T_4_ (FYM application 5 t/ha with gypsum 2 t/ha). The highest effective sodium reduction was observed in treatment T_9_ (RDF+FYM_5_+PM_5_+Gyp_1_) which are 13.1%, 17.9 % and 36.4 % in T_5_, T_7_ and T_9_, respectively. Similar pattern application of soil amendments in this experiment and after analysis of sodium content after one year incubation period and found that application of gypsum in the addition to RDF mediated the sodium content from 627 to 633 mEq/L. Further, the addition of FYM levels (5 and 10 t/ha), PM (5 to 10 t/ha) with gypsum levels 1 and 2 t/ha. Application of PM at 10 t/ha with gypsum at 1 t/ha reduced more (484 *m*Eq/L) sodium than a similar amount of FYM application with gypsum (592 *m*Eq/L). In another treatments, like addition of organic amendment at 5t/ha through FYM, PM and a half of the dose through PM and half from FYM with gypsum at 2 t/ha reduced the sodium content. However maximum reduction in treatment T_8_ >T_6_ >T_4_ were 29.5%, 18.8% and 12.4 %, respectively. The mean of both the time period on sodium content with respect to the treatment was also showed lower down the sodium with the addition of organic and gypsum substances. The treatment T_9_, which had half of the organic matter added by FYM and the rest by PM with 1t/ha gypsum application during the research, had the greatest reduction in sodium content. Pressmud and molasses were utilized as soil additives in the early twentieth century. The importance of pressmud as an organic manure for use in agriculture has long been known, as it contains vital plant nutrients in an organic form while also acting as a powerful soil ameliorant [27] . Sodic soils are characterized by clay complexes with high proportions of sodium (Na^+^) on their exchange and alkaline soil reaction [28] that together have detrimental effects on soil aeration and water movement [29], root development [30], plant seedling emergence and enzymatic activities [31], resulting in poor crop yields [26, 32]. Application of gypsum with the organic soil amendments dispersed the Na^+^ ions and flocculating the Ca on exchange sites and improves the soil health [26].

**Table 2** Effect of FYM, pressmud and gypsum application on sodium content (*m*Eq/L)

| Treatment | Duration | | Mean |
| --- | --- | --- | --- |
|  | 6 Months | 12 Months |  |
| Control | 626^a^ | 627^a^ | 626 |
| RDF | 629^a^ | 633^a^ | 631 |
| RDF+Gyp_1_ | 618^a^ | 590^b^ | 604 |
| RDF+FYM_5_+Gyp_2_ | 598^a^ | 549^c^ | 573 |
| RDF+FYM_10_+Gyp_1_ | 544^b^ | 529^d^ | 537 |
| RDF+PM_5_+Gyp_2_ | 543^b^ | 509^e^ | 526 |
| RDF+PM_10_+Gyp_1_ | 514^b^ | 48^4f^ | 499 |
| RDF+FYM_2.5_+PM_2.5_+Gyp_2_ | 507^b^ | 442^g^ | 475 |
| RDF+FYM_5_+PM_5_+Gyp_1_ | 398^c^ | 384^h^ | 391 |

Note: Treatment means in a column with the letters in common are not significant by Duncan’s New Multiple Range Test at 5% level of significance.

*Effect of soil amendment on available N content*

Nitrogen mineralization kinetics in the soil is very much affected by the addition of organic matter and gypsum. During the experiment, it was observed that nitrogen concentration was almost equal to the T_1_ (180 kg/ha) and T_2_ (191 kg/ha) treatment as measured in T_3_ (190 kg/ha), whereas, the addition of gypsum application with organic soil amendments through FYM and PM enhanced the available N concentration in soil (Table 3). Application of FYM at 10 t/ha improved the available N content from 203 to 218 kg/ha by the addition of gypsum 1t /ha. Comparison month the organic treatment at the 10 ton /ha application with 1 t/ha gypsum application, both the organic amendment performed at par and showed value 218 in T_5_ and 217 in T_7_. The highest available N (230 kg/ha) was reported by the addition of organic matter FYM at 5 t/ha and 5 t/ha PM along the 1 t/ha gypsum application (Fig. 1).

**Fig.1** Effect of different treatments on available N in soil.

At 12 months of the incubation period, mineralization rate of applied the soil organic carbon enhanced by the source and graded application of gypsum. Addition of 5 t/ha FYM with 1 t/ha gypsum enhanced available N 8.8% over control. Among the delivery of organic matter through different sources at 5 t/ha with 2 t/ha gypsum application significantly affected the available N content in soil. Addition of organic matter at 5 t/ha through FYM, PM and 50: 50 ratio of FYM and PM reported available N 20%, 15.3%, 28.2% over to control, respectively. Similar pattern application of 10 t/ha of organic matter through FYM, PM and mixing of an equal amount of PM and FYM along with 1 t/ha gypsum application enhanced the available N 34 %, 34.1 % and 41.2 % higher available N in soil over control, respectively. Here, result showed that FYM and PM both were significantly (0.05%) at par in available N over control. Conjugation of equal amounts (50:50) of FYM and PM enhanced the available N as a single application of organic matter through FYM and PM (each 10 t/ha). The mean available N during the experiment was much affected by the organic sources and graded level of gypsum. Highest available N was measured in treatment T_9_ (RDF + FYM_5_ + PM_5_ + Gyp_1_) 235 kg/ha as compared to control (175 kg/ha), RDF (190 kg/ha), in T_5_ (223 kg/ha) and T_7_ (223 kg/ha).

**Table 3** Effect of FYM, pressmud and gypsum application on soil available N (kg/ha)

| Treatment | Duration | | Mean |
| --- | --- | --- | --- |
|  | 6 Months | 12 Months |  |
| Control | 180^e^ | 170^g^ | 175 |
| RDF | 191^de^ | 189^ef^ | 190 |
| RDF+Gyp_1_ | 190^e^ | 185^f^ | 188 |
| RDF+FYM_5_+Gyp_2_ | 203^cd^ | 204^d^ | 204 |
| RDF+FYM_10_+Gyp_1_ | 218^ab^ | 228^b^ | 223 |
| RDF+PM_5_+Gyp_2_ | 205^bc^ | 196^e^ | 201 |
| RDF+PM_10_+Gyp_1_ | 217^b^ | 229^b^ | 223 |
| RDF+FYM_2.5_+PM_2.5_+Gyp_2_ | 213^bc^ | 218^c^ | 216 |
| RDF+FYM_5_+PM_5_+Gyp_1_ | 230^a^ | 240^a^ | 235 |

Note: Treatment means in a column with the letters in common are not significant by Duncan’s New Multiple Range Test at 5% level of significance.

The addition of organic matter through PM application improves the soil microbial population and diversity enhancing the mineralization kinetics of SOM and mediated available N dynamics in soil [34]. Conversion of raw pressmud into enriched pressmud compost through the composting process was increased the available nutrient content like N, P and K. Application of pressmud compost was increased the crop yield and soil properties than raw pressmud [35]. The organic matter added through PM application stimulation of microbial activity could have increased the partial pressure of carbon dioxide (pCO_2_) in the soil solution [36], lowering soil pH and improving the calcium levels, which enhance the plant nutrient dynamics. Sultana et al. [37] reported that incorporation of PM in the combination of 50 percent MSW compost improved available nitrogen content 2-3 times higher in aerobic conditions. Further, described that increasing the mineralization period improved the concentration and availability of N (NO_3_^-^ ^_^N and NH_4_^+^-N ) in soil.

*Effect of soil amendment on available S content*

Available S content in soil was mediated by the application of organic matter in the soil. At six month time interval, the addition of 5 t/ha FYM with 2 t/ha gypsum at par response with RDF and RDF + gypsum at 1 t/ha, whereas, significant improvement over control treatment (Table 4). Treatment T_5_ (RDF + FYM_10_ + Gyp_1_) showed significant improvement in available S (25.1%) over control, this improvement was higher (33.5%) with the application of PM along with 1 t/ha gypsum. Further supplied the 10 t/ha organic matter through the half by the FYM and remaining by the PM with 1 t/ha gypsum (T_9_) showed at par response as T_7_. A similar pattern, the available S mineralization rate was calculated at one year interval and found that increasing the organic matter dose, type and duration significantly affected the S availability in soil. Treatment T_3_ improved the S content from 15.02 to 18.05 kg/ha (Fig. 2).

**Fig.2** Effect of different treatments on available S in soil.

Whereas, application of 5 t/ha organic matter through FYM and PM with 2 ton/ha gypsum showed at par results. Split application of FYM and PM each 2.5 t/ha with 2 t/ha gypsum (T_8_) showed better availability of S in soil over alone application of FYM (T_4_) and PM (T_6_). The addition of 10 t/ha organic matter through FYM, PM and with the split application (FYM and PM) showed significant improvement in available S 20.65, 21.71 and 24.70 kg/ha. This showed that PM application improves the S content in the soil. The mean value of the experiment showed that the addition of organic matter improved the sulphur was 17.60, 18.09, 18.99, 20.23, 19.15, 21.44, 21.03 and 23.24 kg/ha in T_2_, T_3_, T_4_, T_5_, T_6_, T_7_, T_8_ and T_9_ over control (15.44), respectively. Pressmud and gypsum contain a significant amount of S which improves the soil solution S concentration.

**Table 4** Effect of FYM, pressmud and gypsum application on soil available S (kg/ha)

| Treatment | Duration | | Mean |
| --- | --- | --- | --- |
|  | 6 Months | 12 Months |  |
| Control | 15.85^e^ | 15.02^g^ | 15.44 |
| RDF | 17.94^d^ | 17.26^f^ | 17.60 |
| RDF+Gyp_1_ | 18.12^d^ | 18.05^e^ | 18.09 |
| RDF+FYM_5_+Gyp_2_ | 18.83^cd^ | 19.14^d^ | 18.99 |
| RDF+FYM_10_+Gyp_1_ | 19.81^bc^ | 20.65^c^ | 20.23 |
| RDF+PM_5_+Gyp_2_ | 19.08^cd^ | 19.22^d^ | 19.15 |
| RDF+PM_10_+Gyp_1_ | 21.16^a^ | 21.71^b^ | 21.44 |
| RDF+FYM_2.5_+PM_2.5_+Gyp_2_ | 20.98^ab^ | 21.07^bc^ | 21.03 |
| RDF+FYM_5_+PM_5_+Gyp_1_ | 21.77^a^ | 24.70^a^ | 23.24 |

Note: Treatment means in a column with the letters in common are not significant by Duncan’s New Multiple Range Test at 5% level of significance.

Application of S fertilizer in salt affected soils is a viable procedure to counteract the uptake of unnecessary toxic elements (Na^+^ and Cl^-^), which encourage selectivity of K or Na and the ability of calcium ions to decrease the harmful impacts of sodium ions in plants [38, 39]. The availability of S content in soil significantly increased because PM is a good source of S in soil and the slow release nature of PM as OM resulted in higher residual S availability [15]. Further, microbial decomposition could release essential nutrients for plant uptake when the readily available organic C is from the PM [40]. Sultana et al. [37] predicted the plant nutrient dynamics through a kinetic model and indicated that incorporation of poultry manure and sugar pressmud had a higher capability to supply S to the crops. The combined use of biological (beneficial microbes) and organic materials (e.g., compost and straw) as bio-organic amendments with gypsum has great potential in ameliorating saline soils [41]. Sulfuric acid is created in calcareous soils by the action of a genus of autotrophic bacteria, which reacts with native calcium carbonate to make soluble calcium and lower sodium toxicity [42]. Organic materials from the PM improved the microbial community in the soil and acted as a Na ion mediator [34]. However, it also increases the amount of S in the soil solution [41].

**Conclusions**

Sodic soil has a higher concentration of salt ions, which limits nutrient availability during crop growth. The addition of organic and inorganic substances to soil increases the rate of soil organic matter mineralization and the concentration of plant nutrients in soil solution. In the present experiment, graded level of FYM, PM, and gypsum were applied to sodic soil; and monitored the interaction effect on soil pH, salt content, and accessible plant nutrients such as N and S was computed. The results demonstrated that a six-month time period was associated with a reduced rate of organic matter mineralization and lower plant nutrient availability. After one year incubation period PM showed at par or better availability of available N and S and lowered the soil pH and sodium content in soil. Treatment T_9_ (RDF + FYM_5_ + PM_5_ + Gyp_1_) showed better results with respect to lower the sodium content and enhancing the plant nutrient content (N and S) in soil. The findings of this research will be helpful for managing the sodic soils by the application of PM with graded gypsum for sustainable crop production.

**Statements and Declarations**

**Conflict of Interest**

The authors declare that they have no conflict of interest.

**Data availability:** All data generated or analysed during this study are included in this MS; publishing agency should have right to use for public domain.

**References**

1. Shahne AA, Shivay YS (2021) Soil health and its improvement through novel agronomic and innovative approaches. Front Agron 3 : 680456
2. Abbas G, Rehman S, siddiqui MH, Ali HM, Farooq MA, Chen Y (2022) Potassium and humic acid synergistically increase salt tolerance and nutrient uptake in contrasting wheat genotypes through ionic homeostasis and activation of antioxidant enzymes. Plants 11: 263
3. Mandal AK, Sharma RC, Singh G (2009) Assessment of salt affected soils in India using GIS. Geocarto Intl 24: 437–456
4. Sharma DK, Thimmappa K, Chinchmalatpure AR, Mandal AK, Yadav RK, Chaudhari SK, Sikka AK (2015) Assessment of production and monetary losses from salt affected soils in India. Technical Bulletin. ICAR–CSSRI, Karnal, India
5. Rath K.M, Maheshwari A, Bengtson P, Rousk J (2016) Comparative toxicities of salts on microbial processes in soil. App Environ Microb 82(7): 2012–2020
6. Meena MD, Dotaniya ML, Meena MK, Meena BL, Meena KN, Doutaniya RK, Meena HS, Moharana PC, Rai PK (2021) Maturity indices as an index to evaluate the quality of sulphur enriched municipal solid waste compost using variable byproduct of sulphur. Waste Manage 126:180-190
7. Minhas PS, Saha JK, Dotaniya ML, Saha A, Saha M (2021) Wastewater irrigation in India: Current status, impacts and response options. Sci Total Environ 808:1-17
8. Pipalde JS, Dotaniya ML (2018) Interactive effects of lead and nickel contamination on nickel mobility dynamics in spinach. Intl J Environ Res 12(5): 553–560
9. Sheoran P, Kumar A, Kumar A, Raju R, Sharma R, Parjapat K, Chahal VP (2020) Impact of pressmud application in reclamation of high RSC irrigation water induced soil sodification and sustaining rice (*Oryza sativa*)-wheat (*Triticum aestivum*) production in Indo-Gangetic Plains. Indian J Agric Sci 90: 206–211
10. Meena MK, Yadav BL, Dotaniya ML, Meena MD (2022) Can addition of organic manures mediated sodicity toxicity in mustard cultivation ?. Commun Soil Sci Plant Anal 53(1):77-88
11. Dotaniya ML, Datta SC, Biswas DR, Dotaniya CK, Meena BL, Rajendiran S, Regar KL, Lata M (2016) Use of sugarcane industrial byproducts for improving sugarcane productivity and soil health-a review. Intl J Recyc Org Waste Agric 5(3):185–194
12. Kalaivanan D, Omar Hattab K (2008) Influence of enriched pressmud compost on soil chemical properties and yield of rice. Res J Microbiol 3: 254-261
13. Budiyanto G (2021) The effect of combination of sugarcane pressmud compost and potassium fertilizer on vegetative growth of corn in coastal sandy soil. Food Research 5 (3): 289 – 296
14. Dotaniya ML, Meena VD, Saha JK, Dotaniya CK, Mahmoud AED, Meena BL, Meena MD, Sanwal RC, Meena RS, Doutaniya RK, Solanki P, Lata M, Rai PK (2022) Use of poor quality water for sustainable crop production in changing scenario of climate change. Environ, Develop Sust doi 10.1007/s10668-022-02365-9
15. Kumar S, Meena RS, Singh RK, Munir TM, Datta R, Danish S, Yadav GS, Kumar S (2021) Soil microbial and nutrient dynamics under different sowing environment of Indian mustard (*Brassica juncea* L.) in rice based cropping system . Sci Rep 11:5289
16. Meena AL, Jha P, Dotaniya ML, Kumar B, Meena BP, Jat RL (2019) Carbon, nitrogen, phosphorus mineralization as influenced by type of organic residues and soil contact variation in vertisol of central India. Agric Res 9:232–240
17. Singh SK, Dey P, Singh S, Sharma PK, Singh YV, Latare AM, Singh CM, Kumar D, Kumar O, Yadav SN, Verma SS (2015) Emergence of boron and sulphur deficiency in soils of Chandauli, Mirzapur, Sant Ravidas Nagar and Varanasi districts of eastern Uttar Pradesh. J Indian Soc Soil Sci 63(2): 200-208
18. Singh D, Chhonkar PK, Dwivedi BS (2005) Manual on soil, plant and water analysis. Westville, New Delhi
19. Olsen SR, Cole CW, Watanabe FS, Dean LA (1954) Estimation of available phosphorus in soils by extraction with sodium bicarbonate. US Depart Agric, Circ, vol 939 (p. 19). Washington, DC: US Department of Agriculture.
20. Williams CH, Steinbergs A (1969) Soil sulphur fractions as chemical indices of available sulphur in some australian soils. Aust J Agric Res 10:340-352
21. Subbiah BV, Asija GL (1956) A Rapid procedure for the determination of available nitrogen in soils. Curr Sci 25:259-260
22. Gomez KA, Gomez A (1983) Statistical procedures for agricultural research, 2nd edn. Wiley, New York
23. Kumar S, Meena RS, Jinger D, Jatav HS, Banjara T (2017) Use of pressmud compost for improving crop productivity and soil health. Intl J Chem Stud 5(2): 384-389
24. Hattab O, Natarajan KK, Gopalsamy A (1998) Influence of different organic manures on yield and N use efficiency of rice. J Indian Soc Soil Sci 46: 239-242
25. Prabhavathi N, Ramakrishna Parama VR (2019) Effect of sugar industry solid waste press mud and bio compost on soil physical and chemical properties at different intervals during finger millet crop. J Pharma Phytochem 8(3): 3038-3042
26. Ramaswami PP (1999) Recycling of agricultural and agro-industry wastes for sustainable agriculture production. J Indian Soc Soil Sci 47: 661-665
27. Shahid SA, Zaman M, Heng L (2018) Introduction to soil salinity, sodicity and diagnostics techniques. In Mohammad Zaman Shabbir A. Shahid & Lee Heng. Guideline for salinity assessment, mitigation and adaptation using nuclear and related techniques Cham, Switzerland: Springer Open, pp 1–42. https://doi.org/10.1007/978-3-319-96190-3
28. Nelson PN, Ham GJ (2000) Exploring the response of sugar cane to sodic and saline conditions through natural variation in the field. Field Crops Res 66: 245–255
29. Shaw RJ, Coughlin KJ, Bell LC (1998) Root zone sodicity. In sodic soil: distribution, management and environmental consequences. New York, NY: Oxford University Press, pp 95– 106
30. Saviozzi A, Cardelli R, Di Puccio R (2011) Impact of salinity on soil biological activities: A laboratory experiment. Commun Soil Sci Plant Anal 42: 358–367
31. Sharma DK, Singh A, Sharma PC, Dagar JC, Chaudhari SK (2016) Sustainable management of sodic soils for crop production: Opportunities and challenges. J Soil Salinity Water Quat 8: 109–130.
32. Sheoran P, Basak N, Kumar A, Yadav RK, Singh R, Sharma R, Sharma PC (2021a) Ameliorants and salt tolerant varieties improve rice-wheat production in soils undergoing sodification with alkali water irrigation in Indo–Gangetic Plains of India. Agric Water Manage 243: 106492
33. Sheoran P, Kumar a, Singh A, Kumar A, Parjapat k, Sharma R, Singh RK, Yadav RK (2021b) Pressmud alleviates soil sodicity stress in a rice–wheat rotation: Effects on soil properties, physiological adaptation and yield-related traits Land Degrad Dev 32 (9):1–14
34. Dotaniya ML, Aparna K, Dotaniya CK, Singh M, Regar KL (2018) Role of soil enzymes in sustainable crop production. In: Khudus et al. (Ed) Enzymes in food biotechnology, Springer International. pp 569-589
35. Desmukh RR, Kaswala RG, Kaswala AR (2003) Effect of different industrial wastes on nutrient availability in clayey soils (Typic Chromustert) of south Gujarat. Indian J Agric Environ Bio-Tech 1: 108-115
36. Robbins C.W. 1986. Sodic calcareous soil reclamation as affected by different amendments and crops. Agronomy Journal 78: 916–920.
37. Sultana M, Jahiruddin M, Islam MR, Rahman MM, Abedin MA, Mahmud AA (2021) Nitrogen, phosphorus and sulphur mineralization in soil treated with amended municipal solid waste compost under aerobic and anaerobic conditions. Intl J Recyl Org Waste Agric 10: 245-256
38. Wilson CS, Lesch SM, Grieve CM (2000) Growth stage modulates salinity tolerance of New Zealand spinach (*Tetragonia tetragonioides*, Pall.) and red orach (*Atriplex hortensis* L.). Anna Bot 85(4): 501-509
39. Zaman B, Ali A, Salim M, Niazi BH (2002) Role of sulphur for potassium/sodium ratio in sunflower under saline conditions. Helia 25(37): 69-78
40. Ghulam S, Khan MJ, Usman K, Rehman HU (2010) Impact of PM as organic amendment on physico-chemical characteristics of calcareous soil. Sarhad J Agric 26: 565–570
41. Bello SK, Alayafi AH, Al-Solaimani Abo-Elyousr KAM (2021) Mitigating soil salinity stress with gypsum and bio-organic amendments: a review. Agronomy 11: 1735
42. Ahmed K, Qadir G, Jami AR, Saqib AI, Nawaz MQ, Kamal MA, Haq E (2016) Strategies for soil amelioration using sulphur in salt affected soils. Cercetări Agronomice in Moldova. XLIX (3): 5-16
