# Supplementary material for: Management of plant nutrient dynamics under alkaline soils through graded application of pressmud and gypsum
Source: PLoS One. 2023 Aug 9;18(8):e0288784. doi: 10.1371/journal.pone.0288784 (PMC10411785; doi:10.1371/journal.pone.0288784)
Supplement: Attachment — Submitted filename: Response letter to Editor.doc [file pone.0288784.s002.doc]

Response to PONE-D-23-16195 (Management of Plant Nutrient dynamics under Alkaline Soils through graded application of Pressmud and Gypsum).

| **Correction** | **Reply** |
| --- | --- |
| Academic Editor comments | |
| Thank you for submitting your manuscript to PLOS ONE. After careful consideration, we feel that it has merit but does not fully meet PLOS ONE’s publication criteria as it currently  stands. Therefore, we invite you to submit a revised version of the manuscript that addresses the points raised during the review process. | Thank you for kind words. Manuscript was modified as per the direction of Reviewers. |
| **Reviewer 1** |  |
| I have gone through manuscript (MS) entitled “Management of Plant Nutrient dynamics under Alkaline Soils through graded application of Pressmud and Gypsum,"" and made the necessary corrections in the MS text. The study is very useful to use of organic amendments particularly in the alkaline soil. Please go through the corrections as included in MS. | Agreed; Modified the MS as per your directions |
| **Reviwer 2** | |
| I read “Management of Plant Nutrient dynamics under Alkaline Soils through graded application of Pressmud and Gypsum” with a great interest. It is scientifically written as per the journal guidelines. I recommend minor  revision based on the following points | Thank you for valuable comments |
| 1. Is it a field or laboratory experiment?….indicate in material and method part. | Laboratory experiment, addded in M& M part |
| In results and discussion section, there are different ways of representing the units for the one. Please, unify the written units. For example, ton/ha and t/ha | Corrected |
| Check unit of sodium ions | Checked, it is okay |
| Table 1: You have superscripts (a,b, c). What do they refer? | Added in table subnote |
| Fig. 1, Add the error bars. | Added |
| Fill texture in fig 1 & 2 | Corrected |
| Carefully check reference in text and reference section. | Corrected |
| Comple/clearly the reference 4 in text. | Modified |
| Please, update the introduction and discussion with the recent literature. | Modified |
| Conclusion section should be updated and more clear with significant findings. | Modified |

Changes in MS is in track change mode

Kindly acknowledge the receipt of the same.

Warm Regards

Dr M L Dotaniya
